# Structural characterisation of high-mobility Cd_3_As_2_ films crystallised on SrTiO_3_

**DOI:** 10.1038/s41598-018-20758-7

**Published:** 2018-02-02

**Authors:** Yusuke Nakazawa, Masaki Uchida, Shinichi Nishihaya, Markus Kriener, Yusuke Kozuka, Yasujiro Taguchi, Masashi Kawasaki

**Affiliations:** 1 Department of Applied Physics and Quantum-Phase Electronics Center (QPEC), the University of Tokyo, Tokyo, 113-8656 Japan; 2 RIKEN Center for Emergent Matter Science (CEMS), Wako, 351-0198 Japan

## Abstract

Cd_3_As_2_ has long been known as a high-mobility semiconductor. The recent finding of a topological semimetal state in this compound has demanded growth of epitaxial films with high crystallinity and controlled thickness. Here we report the structural characterisation of Cd_3_As_2_ films grown on SrTiO_3_ substrates by solid-phase epitaxy at high temperatures up to 600 °C by employing optimised capping layers and substrates. The As triangular lattice is epitaxially stacked on the Ti square lattice of the (001) SrTiO_3_ substrate, producing (112)-oriented Cd_3_As_2_ films exhibiting high crystallinity with a rocking-curve width of 0.02° and a high electron mobility exceeding 30,000 cm^2^/Vs. The systematic characterisation of films annealed at various temperatures allowed us to identify two-step crystallisation processes in which out-of-plane and subsequently in-plane directions occur with increasing annealing temperature. Our findings on the high-temperature crystallisation process of Cd_3_As_2_ enable a unique approach for fabricating high-quality Cd_3_As_2_ films and elucidating quantum transport by back gating through the SrTiO_3_ substrate.

## Introduction

Since the topological Dirac semimetal state in Cd_3_As_2_ has been theoretically predicted and experimentally verified^1–3^, a variety of its syntheses, such as by melt growth^1–5^, Cd flux growth^6–9^, chemical vapour transport^10–13^, and chemical vapour deposition^14^ have been reported so far. On the other hand, Cd_3_As_2_ has long been known as a high-mobility semiconductor^15^, and growth of rather thick films, mainly by thermal evapouration and pulsed-laser evapouration, has been reported since the 1970s^16–22^. Following the discovery of the Dirac semimetal state, molecular beam epitaxy (MBE) has been employed to investigate quantum transport phenomena in Cd_3_As_2_ thin films^23–25^. However, crystallinity and flatness of these films are still limited, mainly due to the low temperature (~200 °C) growth necessary for avoiding the revapourisation of Cd_3_As_2_ itself. To overcome this issue, we have recently developed a high-temperature annealing method which allows to fabricate very thin (12 nm ~ 20 nm) Cd_3_As_2_ films which exhibit a clear quantum Hall effect with zero resistance^26^. Here we report the detailed structural characterisation of rather thick three-dimensional (100 nm) Cd_3_As_2_ films annealed at various temperatures and elucidate the evolution of their epitaxial crystallisation and its relation to transport properties.

## Results

High-quality Cd_3_As_2_ epitaxial films are fabricated on (001) SrTiO_3_ substrates by high temperature annealing. The SrTiO_3_ substrates are etched with a buffered hydrofluoric acid by a supplier (SHINKOSHA Co. Ltd). As shown in the x-ray diffraction (XRD) pattern later, the Cd_3_As_2_ film is amorphous just after the deposition at room temperature, necessitating an annealing process for crystallisation. Si_3_N_4_ and TiO_2_ were deposited *in-situ* on the Cd_3_As_2_ film as capping layers, which prevent re-evapouration of the Cd_3_As_2_ film during the high-temperature annealing. This combination of the capping layers is chosen due to the chemical inertness of TiO_2_ against Cd_3_As_2_ even if there is direct contact between two and the mechanical toughness of Si_3_N_4_ covering the whole film. The optimised capping layers enable annealing at temperatures as high as 600 °C, where the vapour pressure of Cd_3_As_2_ becomes increasingly high (~10 Torr at 600 °C^27^). Detailed growth conditions are described in Methods section.

**Figure 1:**
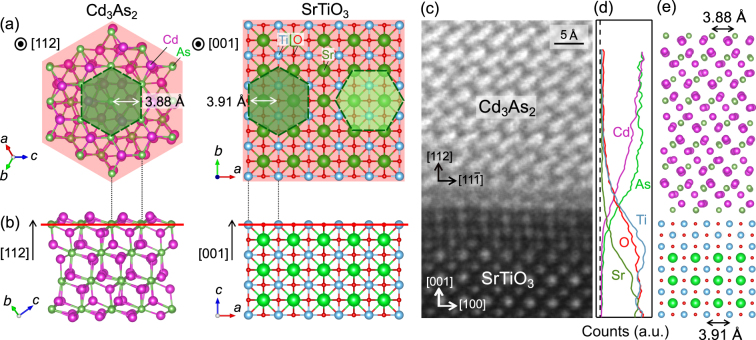
Epitaxial relation between Cd_3_As_2_ and SrTiO_3_. (**a**) Top and (**b**) side views of crystal structures, representing epitaxial [112] Cd_3_As_2_/[001] SrTiO_3_ relation. The green hexagons in (**a**) represent the As triangular lattice in Cd_3_As_2_. There are two possible in-plane alignments on the Ti square lattice (right). (**c**) HAADF-STEM image showing atomic arrangement at the heterointerface between Cd_3_As_2_ film and SrTiO_3_ substrate. (**d**) Depth profile of Cd, As, Sr, Ti, and O, obtained by integrating EDX counts along the horizontal direction in (**c**). Incidentally, Sr EDX counts in the depth profile are certainly suppressed in the interfacial layers as compared to the respective Ti and O results, indicating that a few TiO_2_ layers are formed by high-temperature annealing^29,30^. The STEM and EDX measurements were performed with a low acceleration voltage of 80 kV to reduce the damage at the Cd_3_As_2_ interface, resulting in the lower resolution image compared to those of the central region^26^ taken at 200 kV. The damage is inevitable to some extent, causing deviation from the Cd/As stoichiometric composition at the interface. (**e**) Epitaxial relation of the (112) oriented Cd_3_As_2_ film on the (001) SrTiO_3_ substrate. Projected lattice distances of the triangular-lattice As atoms and the square-lattice Ti atoms are almost the same.

To better understand the epitaxial relation between Cd_3_As_2_ and SrTiO_3_, their lattice structures are presented in Fig. 1. Cd_3_As_2_ forms a solid phase having a cubic Cd-deficient antifluorite (Cd_4_As_2_) structure below 715 °C, and gets successively distorted to form $\sqrt{2}\times \sqrt{2}\times 2$ and 2 × 2 × 4 superstructures below 600 °C and 475 °C, respectively, accompanied with ordered displacements of Cd atoms^28^. In this 2 × 2 × 4 Cd_3_As_2_, a triangular lattice is formed on the (112) lattice plane, which corresponds to the (111) lattice plane of the high-temperature cubic antifluorite structure. The crystal structure of SrTiO_3_ is perovskite type with a square lattice on the (001) plane. The green hexagons in Fig. 1(a) depict the in-plane epitaxial relation between the (112) Cd_3_As_2_ plane and the (001) SrTiO_3_ plane, realizing epitaxial growth of the Cd_3_As_2_ film. The length of the perpendicular line in the As triangular lattice is 3.88 Å (white arrow in the left panel), which is very close to the lattice constant of 3.91 Å in the (001) SrTiO_3_ plane (white arrow in the right panel). Consequently, there are two distinct stacking patterns of the (112) Cd_3_As_2_ plane on the (001) SrTiO_3_ plane, where the [11$\overline{1}$] in-plane Cd_3_As_2_ axis is along either the [100] or [010] direction in SrTiO_3_, as shown in the right panel of Fig. 1(a). Figure 1(c) and (d) show cross-section high-angle annular dark-field scanning transmission electron microscopy (HAADF-STEM) image along with a depth profile of each element obtained by energy dispersive x-ray spectroscopy (EDX) for the Cd_3_As_2_ film annealed at the highest temperature of 600 °C, and a schematic sketch is shown in Fig. 1(e) indicating a possible atomic structure. Incidentally, Sr EDX counts in the depth profile are suppressed in the interfacial layers as compared to the Ti and O counts, indicating that a few TiO_2_ layers are formed at the heterointerface. Such surface termination with a few TiO_2_ layers is known to usually occur when SrTiO_3_ substrates are annealed at such high temperature^29,30^.

**Figure 2:**
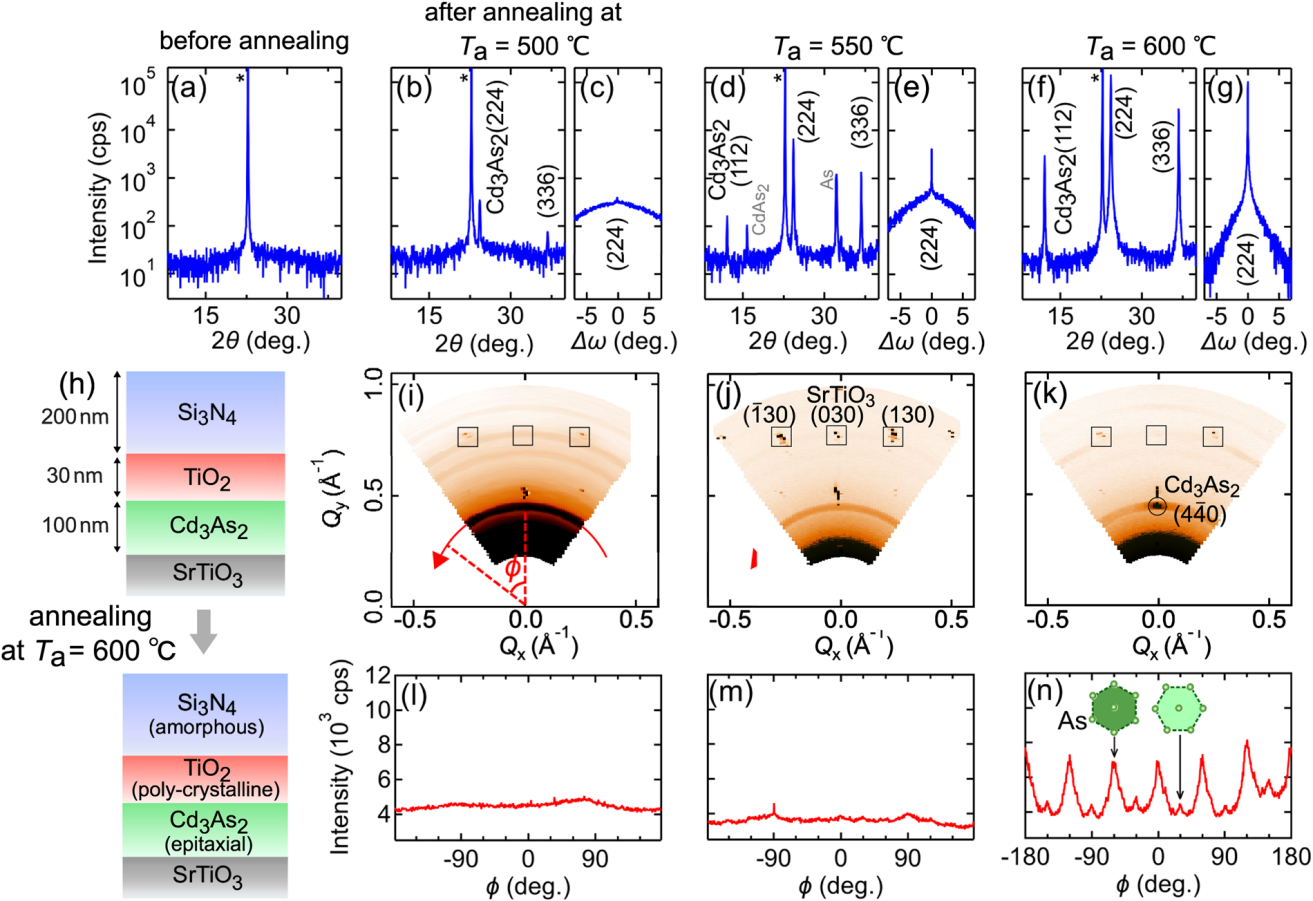
XRD characterisation of the Cd_3_As_2_ films annealed at high temperatures. XRD *θ*-2*θ* scans and corresponding rocking curves of the (224) Cd_3_As_2_ film peaks (**a**) before annealing and (**b**,**c**) after annealing at 500 °C, (**d**,**e**) 550 °C, and (**f**,**g**) 600 °C. (**h**) Sample structure and changes of the crystalline nature in each layer due to the annealing. (**i**–**k**) In-plane reciprocal space mappings and (**l**)–(**n**) *ϕ*-scans along the red curves in the reciprocal space mappings. The *ϕ*-scan pattern shown in (n) represents two sets of 6-fold peaks. Major and minor in-plane stacking patterns of the As triangular lattice are denoted by dark and light green hexagons as shown in the right panel of Fig. 1(a).

XRD *θ*-2*θ* scans and rocking curves of the film peaks are summarised in Fig. 2 for the Cd_3_As_2_ films annealed at various temperatures. The diffraction pattern of the as-grown film shows no film peaks, indicating that the film is amorphous. For the film annealed at 500 °C, weak film peaks assigned to the (224) and (336) plane reflections are observed in the *θ*-2*θ* scan, while a full width at half maximum (FWHM) of the rocking curve for the (224) peak is very broad (9.9°). The *θ*-2*θ* scan for the film annealed at 550 °C shows much stronger peaks originating from the {112} lattice planes, and the FWHM of the rocking curve also became much sharper (0.027°). On the other hand, impurity peaks ascribed to As and CdAs_2_ phases are detected when annealing at this temperature. By increasing the annealing temperature up to 600 °C, a (112)-oriented single-phase Cd_3_As_2_ film is obtained, as shown in Fig. 2(f). The FWHM of the rocking curve is very sharp (0.023°), which is nearly one-fourth of values reported for single-crystalline bulk samples^7^. In both Fig. 2(e) and (f), a rather broad background with weak intensity can be seen (note the logarithmic scale). In case of thinner Cd_3_As_2_ films ($\le$20 nm), this background is not discernible. Presumably, some disorder is present for thicker films away from the Cd_3_As_2_/SrTiO_3_ interface.

In-plane reciprocal space mappings and *ϕ*-scans for the (4$\overline{4}$0) Cd_3_As_2_ peak are presented in Fig. 2(i–n). For the Cd_3_As_2_ films annealed at 500 °C and 550 °C, Debye-Scherrer ring patterns are observed in the reciprocal space mappings and no sharp peaks are confirmed in the *ϕ*-scans. These results indicate that the Cd_3_As_2_ films annealed below 550 °C are not oriented along the in-plane directions, while they are crystallised with the [112] out-of-plane orientation as confirmed in Fig. 2(b) and (d). In contrast, the reciprocal space mapping for the film annealed at 600 °C shows a clear peak from the (4$\overline{4}$0) reflection plane, which exhibits a six-fold symmetry in the *ϕ*-scan. In addition, other six peaks with much weaker intensity are observed between the main peaks in the *ϕ*-scan. The existence of these two sets of peaks with contrasting intensities indicates that there are two types of stacking patterns originating from the epitaxial relation as shown in Fig. 1(a). One of them becomes dominant through the high-temperature annealing, which is possibly due to a miscut direction of the SrTiO_3_ substrates.

**Figure 3:**
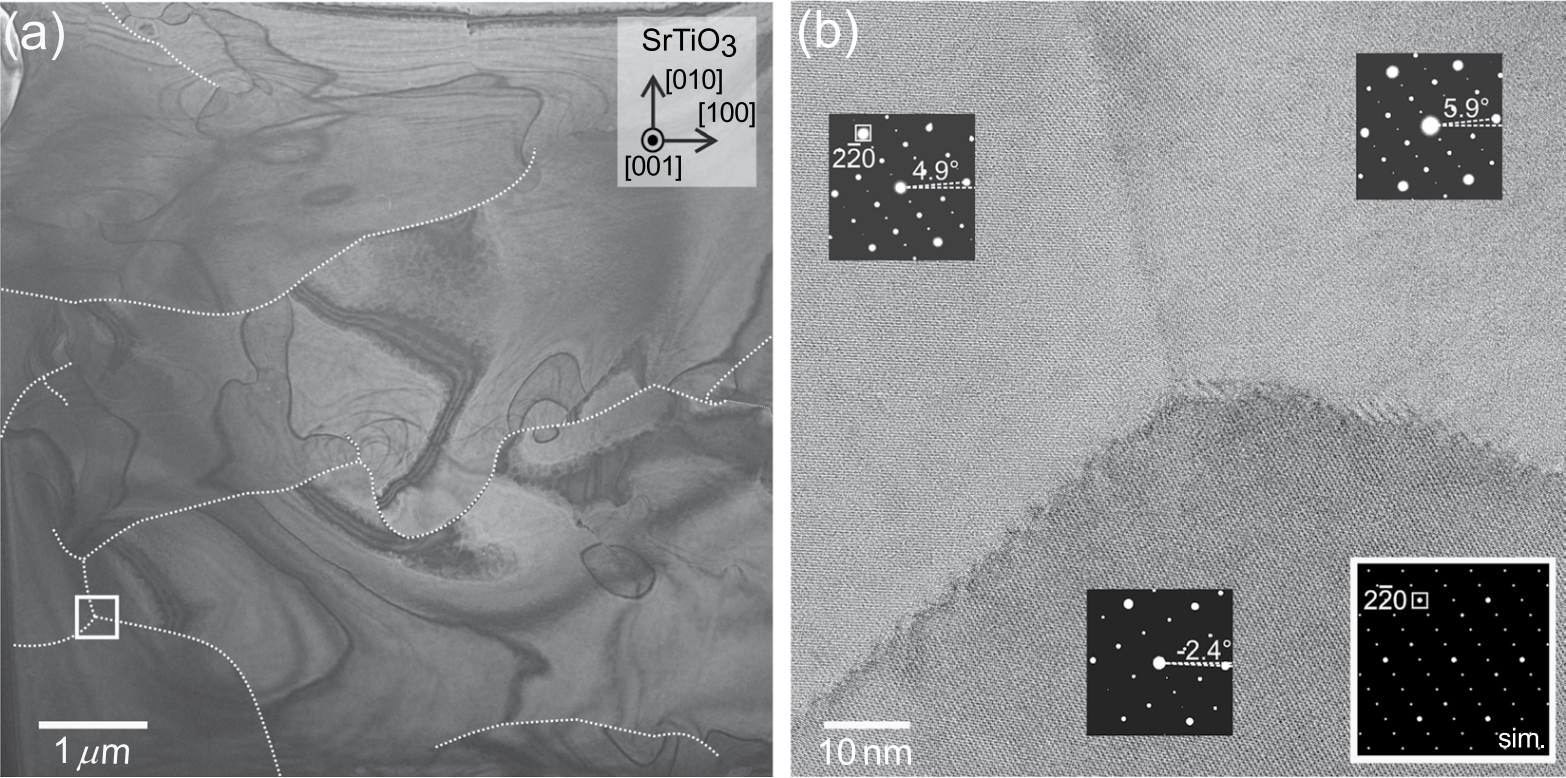
Plane-view TEM characterisation of the Cd_3_As_2_ film. (**a**) Planar TEM picture of the film annealed at the highest temperature of 600 °C. Dotted curves are overlaid on discernible domain boundaries. Crystal axes of the SrTiO_3_ substrate are shown in the inset for reference. (**b**) Higher-resolution magnified image focusing on a tri-sectional grain boundary in the boxed area in (**a**). Electron diffraction patterns taken in the respective domains are shown in the insets. The brightest spot at the center is the zeroth diffraction spot. The diffraction patterns in each grain are slightly tilted as compared to the simulated one (right bottom), where the in-plane crystallographic axes of the Cd_3_As_2_ film are assumed to be completely aligned with the respective SrTiO_3_ axes.

To investigate the in-plane orientation in more detail, a planer TEM image is taken for the film annealed at the highest temperature of 600 °C. Figure 3(a) shows the existence of domains as large as >10 *μ*m^2^. A higher-resolution magnified image of a tri-sectional domain boundary is presented in Fig. 3(b). As shown in the insets, the obtained electron diffraction patterns agree well with the one simulated for the incident beam direction along Cd_3_As_2_ [112]. All these three diffraction patterns correspond to the major stacking pattern of the (112) Cd_3_As_2_ plane depicted as a dark green hexagon in Fig. 1(a), being consistent with the major peaks appearing in in-plane *ϕ*-scan for the Cd_3_As_2_ (4$\overline{4}$0) plane shown in Fig. 2(n). Although these three domains are in the square epitaxial relation, the in-plane orientation exhibits a small variance of several degrees in the tilting angle. This variance of the in-plane orientation also explains the broad peaks observed in the *ϕ*-scan, indicating that the small in-plane misorientation is the origin of the domain formation.

## Discussion

**Figure 4:**
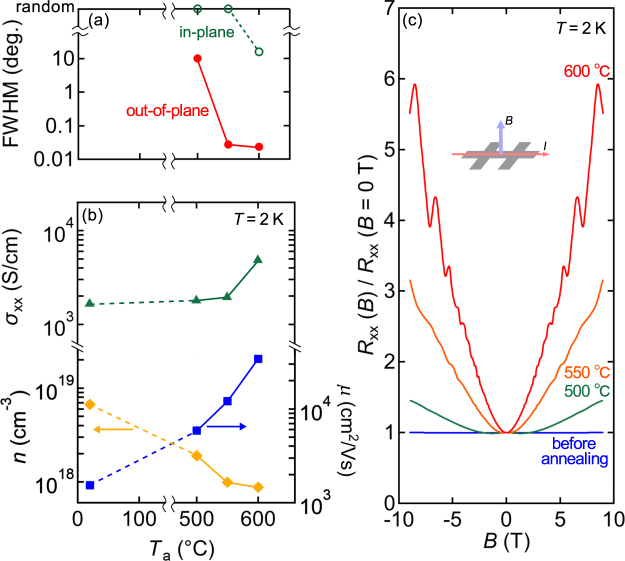
Evolution of crystallinity and transport properties. (**a**) Annealing temperature dependence of the rocking curve width for the out-of-plane (224) Cd_3_As_2_ film peak and the in-plane (4$\overline{4}$0) film peak. (**b**) Same plots for longitudinal conductivity *σ*_*xx*_, carrier density *n*, and the electron mobility *μ* at 2 K. (**c**) Normalised longitudinal magnetoresistance.

Annealing temperature dependences of the film crystallinity and transport properties are summarised in Fig. 4. As detailed in Fig. 2, the FWHM of the rocking curve for the out-of-plane (224) peak sharply drops from 500 °C to 550 °C (red in Fig. 4(a)), whereas annealing at a temperature as high as 600 °C is needed to promote the in-plane orientation alignment (green in Fig. 4(a)). The considerable increase in conductivity occurs between 550 °C and 600 °C, suggesting that the in-plane alignment plays an important role.

The carrier density (*n*, orange) and electron mobility (*μ*, blue) of the Cd_3_As_2_ films for each annealing temperature are deduced from the Hall measurement, as plotted in Fig. 4(b). The carrier density agrees well with one deduced from the SdH oscillations. Before annealing, *n* is 7 × 10^18^ cm^−3^ and it is significantly reduced to 9 × 10^17^ cm^−3^ by annealing at temperatures up to 600 °C. This reduction in *n* may be explained as follows. As shown in the XRD pattern for the film annealed at 550 °C (Fig. 2(d)), arsenic-rich impurity phases (As and CdAs_2_) are confirmed, presumably resulting in arsenic deficiency in the Cd_3_As_2_ phase. Since Cd_3_As_2_ is known to be naturally *n*-type due to As deficiency, *n* of the Cd_3_As_2_ films heated at 600 °C is reduced by the annealing-induced chemical reaction towards a more stoichiometric phase. The mobility *μ* reaches 3.4 × 10^4^ cm^2^/Vs after annealing at 600 °C, while it is 1.5 × 10^3^ cm^2^/Vs before annealing. The increase in the mobility up to the annealing temperature of 550 °C is mainly attributed to the reduction of the carrier density as seen above. The further increase of the mobility from 550 °C to 600 °C is due to the reduction of grain boundaries from in-plane random to epitaxially locked orientations. Figure 4(c) shows longitudinal magnetoresistances of these samples measured with applying the magnetic field perpendicular to the film plane. They are normalised to the zero-field results. With increasing annealing temperature, Shubnikov-de Haas oscillations and quadratic positive magnetoresistance become more pronounced, demonstrating that quantum transport is achievable in films of high-crystallinity and high-mobility.

In summary, we have performed a detailed structural characterisation of high-crystallinity Cd_3_As_2_ films fabricated by high-temperature solid-phase epitaxy. From the systematic characterisation of the films annealed at various high temperatures, successive crystallisation processes take place about the out-of-plane and in-plane orientations. The electron mobility is strikingly enhanced by the epitaxial crystallisation on the square-lattice and the effective reduction of arsenic deficiency. The mobility is expected to be further enhanced by reducing the number of domain boundaries and point defects. Our systematic characterisation of high-quality Cd_3_As_2_ films grown on dielectric oxides provides the foundation to prepare higher-quality Cd_3_As_2_ films by reducing the in-plane domains and carrier densities as well as to investigate quantum transport phenomena by back gating and chemical substitution.

## Methods

Prior to this study, we have screened various materials and tested them for the usefulness as capping layer and substrate. The best results are achieved when using an optimised combination of TiO_2_/Si_3_N_4_ and (001) SrTiO_3_, respectively^26^. For preparing a Cd_3_As_2_ target, 6N5 Cd and 7N5 As shots were mixed at the stoichiometric ratio and heated at 950 °C for 48 hours in a vacuum-sealed silica tube. After heating the mixture, it was grinded and pelletised and then it was sintered at 250 °C for 30 hours in a vacuum-sealed tube. The Cd_3_As_2_ target was ablated using KrF excimer laser at room temperature and a base pressure of about 2 × 10^−7^ Torr. The laser fluence and frequency were set to 0.6 J/cm^2^ and 10 Hz, respectively. Subsequently, 30 nm TiO_2_ and 100 nm Si_3_N_4_ capping layers were deposited by ablating their targets with a laser fluence of 4.0 J/cm^2^ and a frequency of 20 Hz. After all the layers were deposited, the sample was cut into pieces and each pieces was annealed in air at a temperature of 500 °C, 550 °C, and 600 °C for 5 minutes in a rapid thermal annealing system. Annealing at higher temperatures resulted in cracking of the capping layers due to the high vapour pressure of Cd_3_As_2_. Thicknesses of the respective layers were confirmed from Laue oscillations in the XRD *θ*-2*θ* scan.
